# Feasibility and proof-of-concept of a cervical cancer prevention edutainment webisode for African American women in Houston, Texas

**DOI:** 10.3389/ijph.2026.1609804

**Published:** 2026-10-14

**Authors:** Chakema Carmack, Nawar Ahmed, Antonia Marinoni, Terra Ousley, Angelica Roncancio

**Affiliations:** 1 University of Houston Department of Psychological Health and Learning Sciences, Houston, TX, United States; 2 Center for Addictions Research and Cancer Prevention, Health Research Institute, University of Houston, Houston, TX, United States; 3 Psychology Department, University of Houston, Houston, TX, United States; 4 Department of Social Sciences, University of Houston-Downtown, Houston, Houston, TX, United States

**Keywords:** African American women, cervical cancer knowledge, cervical cancer prevention, edutainment, women’s health

## Abstract

**Objectives:**

African American women in Harris County, Texas, have a cervical cancer mortality rate higher than other racial/ethnic minorities. Educational interventions designed to increase women’s participation in cervical cancer screening are needed. The present study examined the feasibility and preliminary proof-of-concept for a culturally tailored cervical cancer prevention edutainment webisode intervention intended to influence knowledge, screening intentions, and HPV vaccination intentions.

**Methods:**

N = 40 African American females were administered a pre-test, completed the edutainment intervention, and completed the post-test 1 week later.

**Results:**

The intervention was feasible regarding recruitment, engagement, retention, and acceptability. Cervical cancer knowledge, susceptibility, and intentions to screen (p <0 .01) showed positive signal detection in the expected direction, indicating proof-of-concept. Most participants found the edutainment film acceptable for various age groups and public health venues.

**Conclusion:**

The intervention demonstrated suitability for future pilot testing and subsequent randomized controlled trial testing to demonstrate efficacy and effectiveness. To mitigate the cervical cancer disparities among African American women, this population should be informed, motivated, and supported through culturally tailored, effective interventions and public health messaging.

## Introduction

In the United States, cervical cancer is the cause of death for over 4,000 women annually [1]. Cervical cancer is primarily caused by long-term infection with high-risk strains of human papillomavirus (HPV). Cervical cancer screening has successfully reduced the incidence and mortality rates of cervical cancer in the United States since the 1940s [2]. Although cervical cancer is highly preventable with regular screenings and the HPV vaccination, disparities persist within underserved populations in the U.S [3].

In the state of Texas, cervical cancer is among the top five most commonly diagnosed cancers for women in their 40s [4]. African American women in Harris County have a cervical cancer mortality of 3.7 per 100,000, higher than their White counterparts (i.e., 2.5 per 100,000) and higher than all other racial/ethnic minorities [4]. Harris County, Texas, which includes the city of Houston and surrounding areas, has been deemed a significant “hot spot” for late-stage cervical cancer diagnoses [5]. This means that women are more frequently diagnosed at advanced stages in Harris County when compared to other regions. These regional cancer cluster areas have a greater impact on cervical cancer mortality for minority women.

Public health and health behavior researchers agree that the most optimal and practical way to prevent cervical cancer morbidity and mortality is to engage in recommended screening behaviors according to established guidelines, such as the U.S. Preventive Task Force (USPTF) [6]. Cervical cancer screening is a broad term that encompasses multiple methods of screening for cervical cancer. These methods include the Papilloma test (i.e., “Pap test” or “Pap smear”), high-risk HPV testing, and co-testing (i.e., a combination of the two methods). The currently accepted guidelines for cervical cancer screening are: a Pap test for 21–65 year olds every 3 years, or high-risk HPV testing alone every 5 years (primary screening) for 30–65 year olds, or co-testing every 5 years for 30–65 year olds [7, 8]. Additionally, the HPV vaccination series is available for males and females (9–26 years old) and is preferred since it is the only primary prevention tool available to help mitigate cervical cancer on a population level [8, 9]. However, sexually active females who have already been exposed to the virus with an active infection, vaccination will not “cure” the active infection, making screening continually imperative [10–12].

### Cervical cancer and African American women

Studies have shown that black women are disproportionately affected by gynecologic cancers such as ovarian, uterine, and cervical cancer [13, 14]. For African American women and other minorities, barriers to prevention include poor health literacy, low education levels, language barriers, socioeconomic status/health insurance status, and distrust of the healthcare system [15–22]. Cultural attitudes, fear, and inadequate communication with healthcare providers also contribute to the differences [17]. Using nationally representative data from the National Health Information Survey, Ford et al. [18] found that African American women were significantly less likely to have heard of HPV, to have reported receiving a Pap screening recommendation by a provider, and less likely to follow up after abnormal results than their White counterparts. Additionally, multiple intersecting social identities (race, gender, and socioeconomic status) shape African American women’s cervical cancer screening experiences [19, 23].

### Edutainment in cervical cancer prevention strategies

Previous studies have shown the effectiveness of various educational interventions designed to increase women’s participation in cervical cancer screening interventions. A qualitative systematic review of empirical intervention studies that targeted adult women, published up to 2018 (N = 37), concluded that these interventions consistently improved cervical cancer knowledge, influenced positive screening attitudes, and increased screening intentions [24]. With the popularity and ease of social media engagement for personal entertainment, it may be worthwhile to implement more *edutainment* interventions to address health behavior outcomes that could be embedded within traditional and social media spaces.

Edutainment is the use of entertainment to educate [25]. Edutainment interventions go beyond traditional educational interventions to utilize elements of entertainment to engage and motivate the target audience. Traditional approaches to cervical cancer prevention emphasize knowledge and facts related to screening guidelines and the severity of cervical cancer. While knowledge and facts are important for preventive health behavior interventions, didactic messaging may be generic and lack relevance if they do not reflect the group’s social context, cultural experiences, or structural barriers. In order for public health messaging to be effective, it must first reach and resonate with the intended target population. Edutainment may use storytelling, games, film, drama, or interactive media to make education enjoyable and relatable. It has been widely used in health promotion, such as cancer prevention, sexual health, and nutrition interventions [26–29]. Digital stories, fotonovela films, and testimonials have been popular modes of cervical cancer prevention edutainment used in intervention research among Hispanic populations [27], while video clips, folk songs, and short films were efficacious among Muslim women [28], for example. While these results were positive and promising regarding edutainment for cervical cancer prevention, they were not tailored for African American women in a specific environmental context (e.g., African American women residing in a large, urban, southern metropolis) as the present study proposed. Culturally tailored edutainment may improve engagement among African American women by solidifying prevention messages through emotional engagement, relevance, and relatable character development. Interactive edutainment serves as an opportunity to incorporate culturally relevant perspectives that can help bridge the gap between knowledge about cervical cancer prevention and motivation toward positive behavior change.

### Information-motivation-behavior model and the Get Your Pap! edutainment webisode

Intervention strategies that are theoretically grounded tend to be most effective in increasing cervical cancer screening rates [24]. The present study used the researcher-developed *Get Your Pap! Edutainment Webisode.* This was a 6-min webisode dramedy film. The webisode was guided by the Information–Motivation–Behavioral Skills (IMB) model [30]. The IMB posits that in order to positively influence preventive health behaviors, individuals must be well informed about the health issue, motivated to act through personal and social influences, and have the behavioral skills necessary to carry out the preventive behavior(s). Information acquisition should provide accurate and relevant knowledge about prevention; motivation would address attitudes, perceived susceptibility, and intentions; and behavioral skills reflect the perceived ability to carry out recommended actions and may also address barriers. The *Get Your Pap! Edutainment Webisode* was designed to deliver culturally relevant cervical cancer information and emphasize perceived susceptibility, which speaks to motivation. The webisode centered around positive attitudes toward screening and modeling an actionable prevention behavior, namely making a cervical cancer screening appointment.

The *Get Your Pap! Edutainment Webisode* featured two African American adult women who were sisters, engaging in a conversation about scheduling their Pap test while waiting for their Uber to arrive. Throughout the webisode, the sisters converse about pertinent cervical cancer information, including education on what it is, the risks for females, the specific risk for African American women, anxiety and misconceptions about the Pap test, and the HPV vaccine. The dynamic is juxtaposed with one sister having a positive, proactive attitude about scheduling her appointment and the younger sister being resistant, initially because she is “not old enough” to be concerned with cancer screening. Specifically, the information component of the IMB was woven throughout the webisode by the younger sister making initial erroneous statements about cervical cancer and the older sister (who is a registered nurse) correcting this misinformation and answering the younger sister’s brief questions throughout their conversation. The motivation component of the IMB was shown through a culturally familiar setting (two sisters chatting in the living room, waiting on their rideshare for an evening out) and relatable dialogue of the sisters. Additionally, personal motivation was shown by the older sister validating and assuaging the younger sister’s concerns with the screening procedures. The behavioral skill component of the IMB focused on self-efficacy in making a screening appointment and normalizing gynecological doctor’s appointments and family conversations. Their interaction plays out in an informative, entertaining, and ultimately heartwarming way.

We employed various research-based entertainment techniques to deliver the cervical cancer prevention messages, including humor (e.g., jokes and common playful slang verbiage), drama (-as the older sister explains a previous cervical cancer scare following an abnormal Pap result), brief animations to keep attention, and other entertainment tactics such as comedically breaking the fourth wall to implement a popular gameshow snippet that requested audience participation about a specific cervical cancer question. The webisode ends with the older sister scheduling her appointment and providing her OBGYN contact info to the younger sister at her request, as the Uber arrives. These intentional elements (a narrative structure and story arc, character experience of an emotional dilemma and decision related to the health behavior, humor, relatable language, and a setting/scenario that reflects the target population), included for the specific purpose of education and behavior modeling, are cornerstones for edutainment. By integrating educational content with culturally resonant messaging that was entertaining, the intervention sought to engage key IMB constructs and produce preliminary changes in knowledge and screening intentions, consistent with a proof-of-concept approach.

### The present study

Taken together, there is a dearth of interventions that address cervical cancer prevention with the use of edutainment and even fewer that are tailored specifically to African American women. The present study was conducted to examine the feasibility (i.e., whether the intervention could be delivered and accepted) and proof-of-concept outcomes (i.e., signal detection; preliminary evidence of the directionality of intervention outcomes) of a brief, culturally tailored cervical cancer prevention edutainment webisode for African American females. We expected that the intervention would show feasibility for implementation. Our proof-of-concept hypotheses were as follows: Following the intervention, participants will a) report greater cervical cancer knowledge, b) report higher intentions to acquire a cervical cancer screening, and c) report higher intentions to complete HPV vaccination.

## Methods

### Participants

Forty African American women (AAW) who resided in the Houston MSA were recruited and enrolled in the study. Eligibility included: (1) self-identify as African American; (2) be of biological female sex; (3) speak and read English; (4) be 18 years old or older; and (5) not currently or previously diagnosed with cervical cancer or cervix dysplasia.

### Webisode and measures

The cervical cancer prevention edutainment webisode information was directly informed by the concept mapping results, previously published [31]. Briefly, the concept mapping procedure was used to graphically model health, behavioral, and cultural information that may increase intentions to screen and screening behavior. Consistent with the IMB model, this “map” was used to identify pertinent information (knowledge), motivation, and skills that needed to be exemplified throughout the premise, scenes, and dialogue within the *Get Your Pap! Edutainment Webisode*. Changes in cervical cancer knowledge supported information components, perceived susceptibility and prevention intentions supported motivational components, and behavioral skills and barriers were addressed through the modeled behaviors of the sisters in the webisode. The research team worked closely with the production company in scripting the webisode vignettes and sequences, ensuring that the film content reflected salient domains from the concept mapping results and contained the information, motivation, and behavioral elements that guided it. The film was vetted for cultural appropriateness and relevancy by three independent researchers with expertise in cancer prevention among minority populations, resulting in a 6-min, live-action film.

All participants were self-identified African American females. Other demographics included 6 items presented in the pretest: age, education, insurance status, and cervical cancer screening history (3 items). Feasibility outcomes were assessed in terms of recruitment, intervention delivery, retention, and acceptability using Orsmond and Cohen’s feasibility objectives and guidelines [32]. Recruitment feasibility was measured by a) the number of women who were screened versus the number of women who were enrolled, b) the eligibility rate, and c) the time to recruit the 40 participants. Intervention delivery was measured by a) the percentage of participants who watched the full webisode (observed with metadata associated with the webisode for each participant) and b) the number of participants who requested to drop out during the project participation. Retention was measured by a) follow-up completion rate at 1 week and beyond, and b) reasons for attrition. Acceptability of the webisode was measured with 7-items on the post-test, indicating favorability of the webisode, benefits of the webisode, and personal feelings about cervical cancer screening after viewing the webisode [32].

Proof-of-concept was evaluated by a single arm, pre-post study design. Proof-of-concept measures included a pre-test and post-survey that reflected common knowledge and understanding previously shown to impact prevention. [8, 31, 33]. The following items were assessed: cervical cancer screening intention (2 items), HPV vaccination intention (2 items), cervical cancer prevention knowledge (10 items), and susceptibility (3 items; Cronbach’s α 0.87). Cervical cancer knowledge was operationalized as an index rather than a psychometric scale. The items assessed true/false cervical cancer facts about what cervical cancer is, risk factors, and screening knowledge. Items were developed based on the USPSTF guidelines to capture distinct cervical cancer prevention knowledge. The guideline information, cervical cancer knowledge, and susceptibility items were directly discussed throughout the sisters’ dialogue in the webisode.

### Procedure

Recruitment of the cervical cancer prevention webisode intervention occurred for 12 weeks to obtain N = 40 African American female adults. Participants were recruited through postings on community bulletin boards in African American communities and churches and at public community events within African American communities. Four community partner organizations also assisted in recruitment. Recruitment materials included physical flyers as well as a digital flyer that included the study title, basic study information, eligibility criteria, and contact information. Community partner organization staff promoted the study through word-of-mouth and flyers provided at their organizations’ resource tables. All participants were recruited and enrolled in the study by the research team at the previously identified recruitment sites. All study procedures were completed online after participant enrollment. The study was reviewed and approved by the university’s institutional review board.

Following enrollment, participants were emailed a unique link to a pre-test survey. Upon pre-test completion, participants were automatically sent a private link to the intervention webisode. After viewing the intervention, they were emailed a unique link to the post-test survey 1 week later. Reminder notifications were automatically sent on the fifth day for participants who had not yet completed the post-test survey (initial reminder notification). Automatic reminders were scheduled to be sent every 3 days following the initial reminder. Following completion of the post-test, participants were emailed a $45 e-gift card for their complete study participation. Additional educational resources were not provided to participants after study completion; however, participants were given referral information for local free/low-cost clinics and health centers within the catchment area. These were displayed at the end of the webisode as well as at the end of the post-test survey. Of note, many of the referral websites provided additional information or reinforced information about cervical cancer and prevention.

### Analysis procedure for proof-of-concept outcomes

To examine proof-of-concept, a single-arm pre-post design was selected. This would allow for the preliminary examination of within-person changes in knowledge, susceptibility, and intentions. It will provide preliminary estimates that can inform the power calculation of a subsequent controlled pilot study to examine efficacy and a randomized controlled trial to examine effectiveness. The present study represented an early-stage proof-of-concept evaluation. For the present study, simple power calculations for comparing paired differences (pre-post) indicated an N = 34 to achieve a power of 80% at an alpha level of 0.05, for detecting a moderate effect size of 0.5. These preliminary results will help to calibrate the expected effect of the observed changes and will be used in sample size calculations needed for controlled trial intervention testing.

Data were screened for outliers and missingness and found to satisfy the assumptions of the statistical tests. Normality for the outcome variables was checked using the Q-Q plot test, which is appropriate for smaller samples. The Q-Q plot showed very minimal, non-substantive deviations from the expected differences in the knowledge, intentions, and vaccine intention outcomes. SPSS v.29 was used for all analyses. Index scores were calculated as the sum of correct responses, consistent with formative measurement approaches in health knowledge assessment. Descriptive statistics were calculated on participant demographics, cervical cancer prevention behavior, and the acceptability and perceived value of the edutainment webisode. Repeated measures t-test served as the primary analysis in evaluating the directionality of the edutainment intervention to increase cervical cancer prevention knowledge. The cervical cancer susceptibility questions were evaluated with the paired-samples proportions test. Researchers were advised through statistical consultation to use the McNemar binomial test statistic, as it is ideal for small sample sizes and paired data. It also corrects for multiple testing beyond the exact binomial test statistic, thereby reducing Type 1 errors. Ad hoc analyses were conducted to investigate potential intergroup trends regarding screening history and intervention acceptability. The *ad hoc* analyses were conducted to characterize sample heterogeneity and identify trends that can be used to inform future intervention refinement and were not meant to test outcomes or confirmatory claims.

## Results

### Demographics and screening history

The study participants included N = 40 African American females. Participants ranged from 21 to 52 years old, and the mean age of participants was 35.7 years (±7.2). All participants self-reported being African American (100%; N = 40) and female gender (100%; N = 40). The educational status of the participants was as follows: 15% (N = 6) high school diploma only; 37.5% (N = 15) some college; 22.5% (N = 9) bachelor’s or technical degree; 17.5% (N = 7) Master’s degree, 2.5% (N = 1) Doctorate; and 5% (N = 2) missing. Regarding insurance status, 70% (N = 28) reported private insurance, 17.5% (N = 7) reported Medicare or Medicaid, 7.5% (N = 3) reported no health insurance, and 5% (N = 2) were missing.

When asked about previous cervical cancer screenings, 50% (N = 20) reported having at least one previous cervical cancer screening, 38% (N = 15) reported not ever having a cervical cancer screening, and 12% (N = 5) reported being unsure. Of the twenty participants who reported having a cervical cancer screening, 55% (N = 11) reported having more than six cervical cancer screenings in their lifetime, 10% (N = 2) reported 5–6 lifetime screenings, 10% (N = 2) reported 3–4 lifetime screenings, 10% (N = 2) reported 2 lifetime screenings, and 15% (N = 3) reported only one cervical cancer screening. Of the fifteen participants who reported not having a cervical cancer screening, 33.3% (N = 5) reported not knowing that it was required for them to get screened, 13.3%% (N = 2) reported that they did not have any symptoms therefore screening was unnecessary, and 53.3% (N = 8) reported that they had not been advised to get screened by their doctor or nurse. Demographics and prevention behavior are shown in Tables 1, 2, respectively.

**TABLE 1:** Sample demographics (Feasibility and Proof-of-Concept of a Cervical Cancer Prevention Edutainment Webisode for African American Women in Houston, TX, United States, 2026).

Demographic	%	N
Female	100	40
Race African American	100	40
Education level High school diploma only Some college Bachelor’s/Technical college Master’s Doctoral Missing	15 37.5 22.5 17.5 2.5 5	6 15 9 7 1 2
Health insurance status Private insurance Medicare/Medicaid No insurance Missing	70 17.5 7.5 5	28 7 3 2
Age	Mean 35.7	S.D. 7.2

**TABLE 2:** Cervical cancer screening behavior (Feasibility and Proof-of-Concept of a Cervical Cancer Prevention Edutainment Webisode for African American Women in Houston, TX, United States, 2026).

Screening behavior	%	N
Cervical cancer screening history (total sample; N = 40) 1 or more None Unsure	50 37.5 12.5	20 15 5
Lifetime cervical cancer screenings (of those with 1 or more previous screenings; N = 20) One only 2 3–4 5–6 More than 6	15 10 10 10 55	3 2 2 2 11
Reported reasons for lack of screening (of those with no previous screenings; N = 15) Did not know it was required Did not have any symptoms/thought screening was unnecessary Had not been advised to get screened by their doctor or nurse	33.3 13.3 53.3	5 2 8

### Feasibility

Feasibility outcomes were assessed in terms of recruitment (threshold N = 40), intervention delivery (threshold >90% of the participants view film completely), retention (threshold >80% retention), and acceptability (threshold >80% favorable). Regarding recruitment, 46 women contacted the study researchers to be screened for study participation. Two potential participants were ineligible due to not self-identifying as African American, and four were ineligible due to having reported a previous diagnosis of cervical dysplasia. This yielded an eligibility rate of 87%. Recruitment lasted approximately 12 weeks. At 10 weeks, 80% (N = 32) of participants were enrolled in the study, and the remaining seven required participants were enrolled in the following 2 weeks. Recruitment ceased upon the enrollment and initiation of study procedures for N = 40 self-identified African American females. All participants (100%) viewed the entire 6-min webisode, and there were no dropouts. Pre- and post-tests took approximately 7 min to complete based on survey metadata. Thus, the intervention showed high intervention delivery and fidelity.

Regarding retention, most participants (78%; N = 31) completed the post-test within 5 days of receiving the post-test link. Reminder notifications were automatically sent on the fifth day for participants who had not yet completed the post-test survey (reminder notification #1). At that time, seven of the remaining nine participants completed the post-test. Automatic reminders were scheduled to be sent every 3 days following the initial reminder (reminder #2). Within 2 days after receiving reminder #2, the remaining two participants completed the post-test survey. Including a 1-week follow-up and two reminder emails sent over 8 days, retention at 2.5 weeks was 100%, exceeding common feasibility benchmarks.

#### Intervention acceptability

Participants were surveyed at post-test only on the acceptability and perceived value of the cervical cancer prevention edutainment webisode. When surveyed about the acceptability of the edutainment webisode, 90% (N = 36) of the participants *strongly agreed* and 10% (N = 4) *agreed* that the webisode would be favorably viewed in women’s health clinics. Ninety-five percent (N = 38) *strongly agreed* and 5% (N = 2) *agreed* that the webisode would be beneficial for 20 and 30-year-olds; and 92% (N = 37) *strongly agreed* and 8% (N = 3) *agreed* that the webisode would be beneficial for 40-year-olds. Furthermore, 87% (N = 35) *strongly agreed*, 4% (N = 2) *neither agreed nor disagreed*, and 7.5% (N = 3) were *not sure* that the webisode would be beneficial for women who are 50 years and older. When surveyed on the perceived value of the cervical cancer prevention edutainment webisode, 87% (N = 35) believed that they knew more about cervical cancer after watching the edutainment webisode, and 80% (N = 32) believed that watching the video put them more at ease about getting their Pap test. After watching the edutainment webisode, 81% (N = 34) of the participants believed that there should be more discourse about cervical cancer within social media spaces. Acceptability metrics of the cervical cancer edutainment webisode are shown in Table 3.

**TABLE 3:** Acceptability of the cervical cancer edutainment webisode (Feasibility and Proof-of-Concept of a Cervical Cancer Prevention Edutainment Webisode for African American Women in Houston, TX, United States, 2026).

Item	%	N
Webisode would be favorably viewed in women’s health clinics Strongly agree Agree Neither agree nor disagree Disagree Strongly disagree	90 10 0 0 0	36 4 0 0 0
Webisode would be beneficial for 20 and 30-year-olds Strongly agree Agree Neither agree nor disagree Disagree Strongly disagree	95 2 0 0 0	38 2 0 0 0
Webisode would be beneficial for 40-year-olds Strongly agree Agree Neither agree nor disagree Disagree Strongly disagree	92 8 0 0 0	37 3 0 0 0
Webisode would be beneficial for women who are 50 years and older Strongly agree Agree Neither agree nor disagree Disagree Strongly disagree	87 4 7.5 0 0	35 2 3 0 0
I Know more about cervical cancer after watching the edutainment webisode Yes No Unsure	87.5 12.5 0	35 5 0
Watching the video put me more at ease about getting their pap test Yes No Unsure	80 0 20	32 0 8
There should be more discourse about cervical cancer within social media spaces Yes No Unsure	81 2.5 12.5	34 1 5

### Intervention proof-of-concept

The pre-test indicated a mean cervical cancer prevention knowledge score of 4.7 (±2.2). The post-test indicated a mean cervical cancer prevention knowledge score of 6.8 (±1.4). Overall, the intervention was successful in increasing cervical cancer prevention knowledge (t(39) = 16.6, p <0 .001). At pre-test, participants indicated a mean intention score to acquire cervical cancer screening of 2.5/5 (±0.3) and 3.9/5 (±0.5) at post-test, indicating a statistically significant increase in their intentions to acquire cervical cancer screening (t(39) = 29.5, p <0 .001). Six participants fell within the age range of 21–26 years old, which is the recommended age for HPV vaccination according to current guidelines. At pre-test, these participants indicated a mean intention score to get vaccinated for HPV of 3.3/5 (±0.1.03) and 4.1/5 (±0.4) at post-test, indicating a statistically significant increase in their intentions to get vaccinated for HPV (t(5) = -2.7, p = 0.02). Table 4 shows the preliminary intervention outcome results.

**TABLE 4:** Intervention (proof-of-concept outcome) results (Feasibility and Proof-of-Concept of a Cervical Cancer Prevention Edutainment Webisode for African American Women in Houston, TX, United States, 2026).

Get Your Pap! edutainment intervention	Pre-test M (s.d.)	Post-test M (s.d.)	t	p-value	Cohen’s d [95% CI]
Cervical cancer knowledge Cervical cancer screening intention HPV vaccine intention	4.7 (±2.2) 2.5 (±0.3) 3.3 (±1.03)	6.8 (±1.4) 3.9 (±0.5) 4.1 (±0.4)	16.6 29.5 2.7	<0.01** <0.01** 0.02*	2.63 [1.96, 3.28] 4.67 [3.58, 5.74] 43 [0.10, 0.75]

*Significant at the .05 level. **Significant at the .01 alpha level.

Regarding cervical cancer susceptibility, when asked, “How many times would you say someone has to have sex to get HPV?” 6% of participants correctly answered *It only takes one time* at pre-test, and 91% of participants answered correctly at post-test, indicating a significant pre-post difference (z(38) = 5.4, p <0 .01). When asked, “African American women are _[blank]_ percentage more likely to die from cervical cancer than some other groups,” 35% of participants correctly answered *75% more likely* at pre-test, and 88% of participants answered correctly at post-test, indicating a significant pre-post difference (z(38) = 3.8, p <0 .01). When asked, “About what percentage of cervical cancer is preventable in African American women,” 17.6% of participants correctly answered *About 94% preventable* at pre-test, and 65% of participants answered correctly at post-test, indicating a significant pre-post difference (z(38) = 4.0, p <0 .01). Table 5 shows the susceptibility results.

**TABLE 5:** Cervical cancer susceptibility results (Feasibility and Proof-of-Concept of a Cervical Cancer Prevention Edutainment Webisode for African American Women in Houston, TX, United States, 2026).

Cervical cancer Susceptibility	Pre-test proportion	Post-test proportion	z	p-value	Cohen’s D [95% CI]
How many times would you say someone has to have sex to get HPV? *It only takes one time*	0.06	0.91	5.4	<0.01**	0.85 [0.49, 1.21]
African american women are _[blank]_ percentage more likely to die from cervical cancer than some other groups? *75% more likely*	0.35	0.88	3.8	<0.01**	0.60 [0.26, 0.94]
About what percentage of cervical cancer is preventable in african american women? *About 94% preventable*	0.18	0.65	4.0	<0.01**	0.63 [0.29, 0.97]

** Significant at the .01 alpha level.

The sample consisted of a relatively well-educated group of African American females (>70% reported some college or higher education degrees) and mostly insured (88% insured). However, because cervical cancer screening is typically recommended until age 65, there was a wide range of ages. Therefore, *ad hoc* analyses were conducted to examine whether screening history and reasons for not getting screened were associated with age. There were no significant differences between those who reported at least one previous screening and those who did not. However, among the fifteen women who reported having no screening history, significant differences in their average age were observed (F(2,12) = 5.3, p = 0.02). In particular, those who reported not knowing it was required were significantly younger (∼28 years old) than those who reported it not being advised by their doctor or nurse (∼40 years old). Table 6 shows the age associations with screening history and reasons for lack of screening.

**TABLE 6:** Reasons for lack of screening associations with participant age (Feasibility and Proof-of-Concept of a Cervical Cancer Prevention Edutainment Webisode for African American Women in Houston, TX, United States, 2026).

Reasons for lack of screening (N = 15)	N	Mean age (s.d.)	F	p-value
Did not know it was required No symptoms/thought screening was unnecessary Not advised by doctor/nurse	5 2 8	27.8 (±6.1) 31.5 (±9.2) 39.8 (±5.4)	5.3	0.02*

* Significant at the .05 alpha level.

## Discussion

The present study tested whether a short, culturally tailored, live-action, edutainment film was feasible and showed proof-of-concept for signal detection toward improving cervical cancer prevention knowledge and screening intentions among African American women in Houston, TX. This adds to the literature that supports the development of culturally tailored cervical cancer prevention edutainment interventions [25, 26]. Similar to Weschasat et al. [28] and Thompson et al. [27], the present study showed that culturally relevant edutainment interventions show positive signal detection for increasing cervical cancer knowledge in underserved populations such as African American females.

Overall, feasibility benchmarks for recruitment, intervention delivery, retention, and acceptability were met, supporting the potential for a controlled pilot study and subsequent randomized controlled trial. The present study was concerned with carrying out the intervention as intended (i.e., feasibility) and examining signal detection. A controlled pilot trial will be concerned with refining effect size estimates and demonstrating efficacy. A subsequent randomized controlled trial will be concerned with demonstrating effectiveness. The present study does not claim efficacy or effectiveness; only that feasibility and proof-of-concept for signal detection were sufficiently demonstrated.

The proportion of women who had not been previously screened was consistent with prior studies focusing on underserved populations [34–37], indicating that the intervention was reaching its target population. The significant age difference in reasons for non-screening was conceptually coherent, given that younger women may be more concerned with access, time, and perceived invulnerability, while older women may be more reliant on doctors’ authority and direct advice. Nevertheless, it is useful data for future intervention refinement and examination that addresses unique barriers that may vary by age and developmental stage for African American women.

Signal detection regarding the pre-post increases in cervical cancer knowledge echoes previous studies that found positive outcomes in the expected direction [37, 38]. Large effect sizes for changes in knowledge and intentions, and moderate-to-large susceptibility effect sizes were observed. However, the pre-post outcomes were powered for signal detection and not calibrated for intervention efficacy, effectiveness, or impact. Therefore, the large effect sizes should be taken with caution as they tend to be unstable in small feasibility studies [39]. There is the potential for overestimation of the observed effect size with small samples. In other words, random sampling variation can produce substantially larger effect sizes than would be observed in the true underlying population. Neither intervention efficacy, effectiveness, nor impact can be established in the present study. Rather, these findings imply a substantive signal detection and not statistical effectiveness.

### Research and practice implications

There are several research implications provided through the study. The high completion and retention implied procedural feasibility, and this supports the progression to controlled pilot testing. The use of the IMB framework was supported through the observed increases in knowledge and will be useful for conservative future trial planning. The relatively large pre-post changes in knowledge, intentions, and susceptibility indicated a positive directionality, although we are careful not to imply efficacy without further testing. Given the feasibility results, it is reasonable to investigate these findings within a controlled design, as the results will inform conservative planning.

Edutainment may help reduce feelings of anxiety in African American women about getting screened, as the majority of the sample reported being more at ease after watching the webisode. This also demonstrates the need to demystify screening misconceptions and improve screening rates [33, 40]. The results support that African American women may benefit from the webisode being shared on social media [41], given that participants reported wanting more social media discourse related to cervical cancer awareness.

Demonstrating edutainment feasibility in a population with historically lower prevention behavior addressed a critical gap in cervical cancer prevention research. The webisode intervention showed promise for low-burden dissemination because the intervention was entertaining by design and can be implemented in a short time duration, when the participants prefer, given that they have a personal computing device (e.g., laptop, tablet, or cell phone). After production, the brief webisode format minimizes cost and staff burden, supporting future implementation research. This suggests potential for scalability, particularly beneficial in community-based, resource-constrained settings. In partnership with cervical cancer prevention services and digital health platforms, the webisode intervention may have the potential to reach an audience that may not have access to a provider’s advice about preventing cervical cancer.

The lack of cervical cancer prevention knowledge, along with reporting not being advised by a doctor to get screened, reflects gaps in communication, trust, and access within healthcare systems that negatively influence cervical cancer outcomes in this population [42, 43]. Thus, there is a significant impact of a lack of provider communication on preventive care that the healthcare system has yet to fully address. This may emphasize the need for tools like edutainment webisodes that help educate African American women, if found successful through a subsequent randomized controlled trial. Utilizing edutainment tools for community outreach shows promise to help bridge knowledge gaps, build trust, and influence health behavior.

Globally, cervical cancer remains one of the leading causes of cancer-related mortality among women, and global health initiatives increasingly emphasize the elimination of cervical cancer as a public health problem [44]. Limited prevention knowledge and other structural barriers contribute to poorer outcomes worldwide, not just among U.S. sub-populations [45]. Incorporating such approaches globally could complement existing public health strategies by reaching women in resource-constrained settings, normalizing conversations about cervical cancer, and empowering women to seek preventive care [46]. Innovative, low-burden, and culturally relevant communication tools may serve as vital complements to screening programs [47].

### Limitations and future research

The present study was conducted as a feasibility study with proof-of-concept outcomes; thus, its scope was limited. Limitations of the present study include a small sample size, which may contribute to the overestimation of the effect size, and the lack of a randomized controlled group. Thus, neither efficacy, effectiveness, nor impact can be established at this time. Although the sample size was sufficient for a feasibility and proof-of-concept study, the present study cannot be generalized beyond the sample or seen as a reflection of all African American women. Participants were obtained specifically from the catchment area of three underserved super neighborhoods in Houston, Texas. Although considered underserved low-resource areas, the current sample consisted of relatively high education levels and health insurance. Therefore, the present study does not reflect the many diverse backgrounds and lifestyles of African American women in that area and limits external validity. Participants with greater educational attainment may find the cervical cancer workshop and information easier to understand. Psychosocially, it may be easier for participants with consistent health insurance to form screening intentions due to fewer financial and access barriers. Homogeneity across these characteristics may have influenced the observed feasibility and effect size results. Education, insurance status, and other locales may differ in potential participants’ self-selection to participate in such an intervention. Nevertheless, the positive signal detection and statistical significance imply a non-zero difference in the expected direction and warrants further investigation. Although knowledge was retained at the 1-week follow-up, this does not imply long-term knowledge, as our ultimate goal is for women to build a habit of obtaining recommended screenings.

Because multi-component approaches such as combining educational films with group discussions or printed materials tend to be more effective than interventions with only one educational component [24], the present study is limited by only one component (i.e., the webisode). Although the webisode’s components were guided by the IMB model that specifies attitudes, norms, and susceptibility as indicators of motivation, the present study only measured susceptibility as a proxy for motivation; nor did it measure actual behavior (e.g., appointment setting and screening completion). Many prevention theories specify that behavior is influenced by the weight of intentions. However, intentions may not always translate into actual behavior, and this is known as the intention-behavior gap. The intention-behavior gap, particularly for cancer screening, has been documented in previous research showing that intentions accounted for approximately 30% of cancer screening variance [48].

Future research will be concerned with refining the intervention for a pilot trial and subsequent randomized controlled trial. While a feasibility study is concerned with whether the intervention can be delivered as intended, and the proof-of-concept outcomes identify signal detection, a subsequent pilot study will examine whether a definitive trial can be executed as designed and will include a control group, randomization, and behavioral outcome follow-ups. Project refinement will include attitude and social norms measurements for the pilot trial and will allow for the examination of specific intervention components that had the greatest effect on knowledge and behavior change. Future research will also include a longer follow-up latency and the outcome of whether participants actually made a screening appointment and completed cervical cancer screening within 6 months. The large effect sizes observed in the present study will be down-weighted by at least one-third (potentially one-fourth) to establish more stable and accurate estimates. Recruitment of the future pilot trial will intend to obtain enough African American females ages 18–26 who fall within the recommended HPV vaccination guidelines. While African American females as a racial group are largely affected by cervical cancer disparities, the subgroups experiencing the greatest structural barriers to prevention behavior and healthcare services are typically less educated and uninsured. We intend to expand our recruitment efforts to reach a more heterogeneous group of African American women with regard to education and insurance status. We intend to power our subsequent studies to be able to detect group differences, allowing us to examine differences in intervention outcomes based on subgroups (e.g., differences and trends related to education level). Lastly, future research should also examine multicomponent strategies that can improve intervention outcomes (e.g., reminders for making a screening appointment) [49]. Nevertheless, the findings show promise but will require a more robust examination with a comparison group, greater sample variability regarding access indicators (e.g., education and insurance status), and a larger sample size.

### Conclusions

To mitigate cervical cancer health disparities among African Americans, posting guidelines alone is not enough. Public health messages and screening guidelines may not reach their intended audience. If they are lacking in relevance, the message may be lost. This present study demonstrated the potential of a brief edutainment webisode intervention to positively influence cervical cancer knowledge and intentions. However, screening uptake, as a prevention behavior outcome, must be measured in a subsequent pilot trial to establish a tangible impact. The solution to ameliorating cervical cancer disparities requires actively working towards acknowledging, understanding, and resolving the disparities at each point on the cancer pathway [3]. The findings support further rigorous trial testing of edutainment in the form of a live-action webisode in addressing cervical cancer prevention among African American women. More research and implementation are crucial for efforts to develop evidence-informed, sustainable, best-practice interventions culturally tailored to this population.

## Data Availability

Aggregated data supporting the conclusions of this article may be made available by the authors on request.
